# Silibinin effects on cognitive disorders: Hope or treatment?

**DOI:** 10.22038/AJP.2023.21959

**Published:** 2023

**Authors:** Zahra Akhoond-Ali, Alireza Rahimi, Atiyeh Ghorbani, Fatemeh Forouzanfar, Sara Hosseinian, Hamed Ghazavi, Farzaneh Vafaee

**Affiliations:** 1 *Neuroscience Research Center, Mashhad University of Medical Sciences, Mashhad, Iran *; 2 *Department of Neuroscience, Faculty of Medicine, Mashhad University of Medical Sciences, Mashhad, Iran*; 3 *Material Science and Metallurgy Engineering, Karaj Branch, Islamic Azad University, Karaj, Iran*; 4 *Department of Physiology, Faculty of Medicine, Mashhad University of Medical Sciences, Mashhad, Iran*; 5 *Applied Biomedical Research Center, Mashhad University of Medical Sciences, Mashhad, Iran*

**Keywords:** Silibinin, Cognitive disorders, Neuroinflammation, Oxidative stress

## Abstract

**Objective:**

Almost all diseases of the nervous system are related to neuroinflammation, oxidative stress, neuronal death, glia activation, and increased pro-inflammatory cytokines. Cognitive disorders are one of the common complications of nervous system diseases. The role of some plant compounds in reducing or preventing cognitive disorders has been determined. Silibinin is a plant bioflavonoid and exhibits various effects on cognitive functions. This article discusses the different mechanisms of the effect of silibinin on cognitive disorders in experimental studies.

**Materials and Methods:**

Databases, including ISI, , Google Scholar, Scopus, Medline and PubMed, were investigated from 2000 to 2021, using related keywords to find required articles**.**

**Results:**

Silibinin can improve cognitive disorders by different pathways such as reducing neuroinflammation and oxidative stress, activation of reactive oxygen species- Brain-derived neurotrophic factor- Tropomyosin receptor kinase B (ROS–BDNF–TrkB) pathway in the hippocampus, an increase of dendritic spines in the brain, inhibition of hyperphosphorylation of tau protein and increasing the expression of insulin receptor (IR) and insulin-like growth factor receptor 1 (IGF-1R), inhibiting inflammatory responses and oxidative stress in the hippocampus and amygdala, and decrease of Homovanillic acid/Dopamine (HVA/DA) ratio and 3,4-Dihydroxyphenylacetic acid + Homovanillic acid/Dopamine (DOPAC+ HVA/DA) ratio in the prefrontal cortex and 5-hydroxyindoleacetic acid/5-hydroxytryptamine (5-HIAA/5-HT) ratio in the hippocampus.

**Conclusion:**

These results suggest that silibinin can be considered a therapeutic agent for the symptom reduction of cognitive disorders, and it acts by affecting various mechanisms such as inflammation, programmed cell death, and oxidative stress.

## Introduction

Cognitive impairment directly impacts a person's ability to live independently and is a crucial determinant of quality of life. Some cognitive functions are age-dependent; for example, with increasing age, a significant decrease in functions such as attention, working memory, executive function, and verbal recall occurs (McDonald, 2017 ▶). However, some cognitive disorders are secondary complications of diseases that affect the nervous system (Pardridge, 2012 ▶). 

Due to the nervous system's low ability to regenerate cells, some cognitive impairments may remain for the whole life and turn the person from an influential person in society to a sick and burden. Drugs that are applied for cognitive disorders have different efficacies in different individuals with different types of cognitive disorders and, in addition to their beneficial effects, have inevitable side effects (Patel, 2016 ▶; Akram and Nawaz, 2017 ▶; Chutko and Surushkina, 2021 ▶). Since natural products offer potential opportunities to discover effective compounds for developing new drugs, using effective compounds in plants is one of the therapeutic goals for cognitive disorders. In this regard, compounds with different properties have been studied. Some of these compounds are used to improve lifestyle and prevent diseases, while others are used as medicine to treat diseases (Iriti et al., 2010 ▶; Parvez, 2017 ▶).

Common events in almost all nervous system diseases include neuroinflammation, oxidative stress, neuronal death, activation of the glia, and increased pro-inflammatory cytokines (Akram and Navaz, 2017 ▶; Fernandes et al., 2018 ▶; Kim et al., 2017 ▶; Liu et al., 2021 ▶; Song et al., 2016 ▶, 2017). Therefore, herbal compounds that have a therapeutic effect on many of these disorders can be more helpful in improving or preventing the progression of cognition diseases.

**Figure 1:**
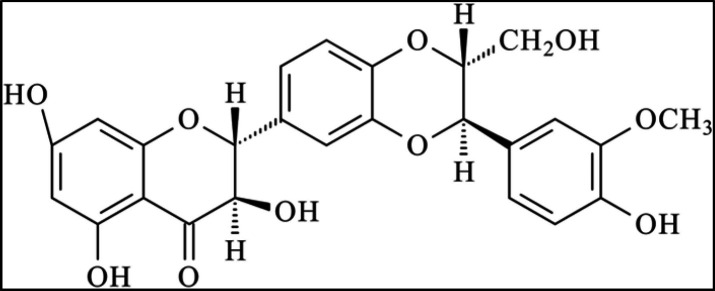
Chemical structure of silibinin

Silibinin is a plant bioflavonoid with molecular formula C25H22O10 (Figure 1) (Ting et al., 2013 ▶; Bijak, 2017 ▶). It is found in silymarin or milk thistle *Silybum marianum* extract. This plant extract contains silibinin, isosilibinin, silychristin, isosilychristin, silydiani, and a flavonoid (taxifolin) (Kim et al., 2003 ▶, 2017). Silibinin has the highest concentration among these compounds and is silymarin's most important biological compound. In addition, there are several pharmacological properties, including antioxidant effects on the CNS and other organs shown by *in vitro* and *in vivo* studies (Marrazzo et al., 2011 ▶; Prabu and Muthumani, 2012 ▶; Jiang et al., 2016 ▶; Federico et al., 2017 ▶; Taleb et al., 2018 ▶; Cho et al., 2021 ▶; Kollaras et al., 2021 ▶). The protective effects of silibinin on the liver have been identified and ascribed to its antioxidant and anti-inflammatory properties (Kollaras et al., 2021 ▶). The antineoplastic effect of silibinin *in vitro* and *in vivo* models of prostate, bladder, skin, clone, breast, lung, and kidney cancers has also been identified (Yassin et al., 2021 ▶).

The effects of silibinin on renal toxicity in rats have shown that it reduces lipid peroxidation and increases the level of the renal antioxidant defense system (Prabu and Muthumani, 2012 ▶). At the molecular level, silibinin reduces inflammatory responses by inhibiting the nuclear factor erythroid 2–related factor 2 (Nrf2) and nuclear factor kappa-light-chain-enhancer of activated B cells (Nf-κB) signaling and suppressing the production of inflammatory cytokines, especially tumor necrosis factor alpha (TNF-α) (Prabu and Muthumani, 2012 ▶; Gupta et al., 2014 ▶). The anti-inflammatory effects of silibinin on H-pylori-induced gastric infection and microglial cell inflammation have been identified by suppressing NF-κB and signal transducer and activator of transcription 3 (STAT3), inhibiting nitrite production and reducing the expression of cyclooxygenase-2 (COX-2) and inducible nitric oxide synthase (iNOS) (Cho et al., 2021 ▶). Therapeutic effects of silymarin on diseases of the nervous system and cognitive complications have been studied (Wang et al., 2012 ▶; Song et al., 2016 ▶; Kim et al., 2017 ▶; Fernandes et al., 2018 ▶; Garikapati et al., 2018 ▶; Masoumi Qajari and Khonakdar-Tarsi, 2021 ▶; Liu et al., 2021a ▶). 

This paper discusses mechanisms underlying the effect of silibinin on cognitive disorders shown by experimental studies.

## Materials and Methods

Online literature resources were checked from 2000 to 2021 using ISI, PubMed and Medline, Google Scholar and Scopus to retrieve articles about the effects of silibinin on neuroprotection. For this aim, keywords in the search were "Silibinin" OR "Silybin" AND "Cognitive disorders" OR "Memory" OR "Learning" OR "Decision making" OR "Addiction" OR “Anxiety" OR "Stress" OR "Depression."

## Results

Cognitive impairment is widespread and can increase with age or occur due to other diseases. Patients who experience various degrees of cognitive impairment report symptoms such as memory loss, attention deficit disorder, decision-making disorder, anxiety, and depression (McCollum and Karlawish, 2020 ▶). As shown in Table 1, silibinin has been helpful as a therapeutic tool to control and improve cognitive disorders. Moreover, silibinin has reduced the oxidant factors and increased the antioxidant power (Shivani et al., 2017 ▶; Taleb et al., 2018 ▶; Liu et al., 2021a ▶). Therefore, silibinin has improved memory impairment and neurogenesis by reducing oxidative stress and inflammation.

Silibinin improves cognitive impairment by reducing the expression of inflammatory factors such as interleukin 1 beta (IL-1β) and IL-6 and suppressing the phosphatidylinositol 3-kinase/protein kinase B (PI3K/Akt) pathway (Jin et al., 2016 ▶; Song et al., 2017 ▶, 2018). In addition, it reduces iNOS and COX-2 and oxidant factors such as malondialdehyde (MDA) levels in the brain and prevents lipid peroxidation by increasing superoxide dismutase (SOD) activity (Shivani et al., 2017 ▶; Song et al., 2017 ▶; Liu et al., 2021a ▶). Table 2 shows the summarized mechanisms of action of silibinin. 

**Effects of Silibinin on learning and memory impairment**

Neuroinflammation is a pathological symptom that occurs in various diseases and can affect cognitive functions, including memory (Eikelenboom et al., 2010 ▶). Lipopolysaccharide (LPS) is a bacterial factor that causes cognitive impairment in animal models by activating the innate immune response (Qin et al., 2007 ▶). Suppression of the inflammatory response is one of the most important methods of controlling inflammation. 

**Table 1:** Effects of silibinin on the cognitive disorder in experimental models

**Ref.**	**Results**	**Silibinin treatment**	**Study design**	**Model**
(Song et al., 2016 ▶)	↑Working memory ↑Spatial memory ↑Learning ↓Neuroinflammation ↓Neuronal Loss	Oral gavage once a day (25, 50, and 100 mg/kg) for 30 days	Male Sprague–Dawley rats (weight 240–260 gr)	Stress and Memory Impairment
(Joshi et al., 2014 ▶)	↑Working memory ↑Spatial long-term memory ↓Anxiety ↓Stress oxidative in HIP and PFC	Oral gavage (50, 10, and 200 mg/kg) daily for seven days	Adult male Wistar albino rats (180–220 gr)	PTSD
(Tota et al., 2011 ▶)	↓Memory impairment	Oral administration (100 and 200mg/kg) for 18 days	Male Swiss albino mice (25–30 gr)	
(Lee et al., 2020 ▶)	↓Depression ↓ Anxiety	25, 50, and 100 mg/kg-IP	Male Sprague Dawley rats, eight weeks old, 220~350 gr	
(Garikapati et al., 2018 ▶)	↑Memory function	Oral administration daily for 40 days (0.01, 0.1, 1 mg/kg)	Male Swiss albino mice (3 months old, weight 20-30 gr)	Chronic stress
(Lu et al., 2010 ▶)	↓Learning and memory impairment	Once daily for seven days	ICR male mic, six weeks old	Addiction
(Wei et al., 2022 ▶)	↑Spontaneous alternation behavior ↑Spatial memory	Gavage, daily for three weeks **(**100, 200 mg/kg)	Adult C57 mice	cognitive impairment
(Gu et al., 2018 ▶)	↑Learning and memory ↑BBB integrity	1,100 mg/ml	Female hamsters, 14 months old, weigh 150–200 gr	Dementia induced-familial hypercholesterolemia
(Wang et al., 2012 ▶)	↓Brain edema ↓Infarct volume	Intragastric administration of silibinin (50, 100 mg/kg) 30 min before pMCAO	Male Sprague-Dawley rats (weight 250–300 gr)	Ischemic stroke
(Xie et al., 2014 ▶)	↓Neuronal cell death ↓Neuronal cells apoptosis	Dissolution of Silibinin in DMSO (1–200 µM) in the culture medium	Cell culture (mouse cortical neurons)	Ischemic stroke Oxidative-nitrosative damage and astrocyte activation
(Fernandes et al., 2018 ▶)	↓Astrocytes activation ↓Oxidative stress ↓Nitrosative stress	(12.5, 25, and 50 μM) for 24 hrs on the cover slide	Cell culture (C6 astrocytoma cells line)	
(S. Kim et al. 2017 ▶)	Delay in the onset of a seizure ↓Frequency of chronic spontaneous seizure ↓GCD ↓Neuronal cell loss ↓Apoptosis ↓Autophagy ↓Neuroinflammation	ip injection (50, 100, 200 mg/kg) 1-35 days	Male C57BL/6 mice (8-week-old, 20~25 gr)	Epileptic seizures
(Jin et al., 2016 ▶)	↓Memory deficits ↓Microglial activation ↓Neuroinflammation ↓Neuronal death	Administration of oral silibinin (100 or 200 mg/kg) once per day for six consecutive weeks	SAMP8 and SAMR1 male mice (26±2 gr, six months	Alzheimer’s disease (AD
(Song et al., 2018 ▶)	↓Learning and memory impairment	Injection of silibinin (25, 50, and 100 mg/kg) into the hippocampal CA1 region	Male Sprague–Dawley rats (220–260 gr)	
(Shen et al., 2019 ▶)	↓Memory deficits ↓Amyloid plaque in HIP	Gavage (100 mg/kg) once a day for 15 days	Male APP/PS1 mice (7 months old)	
(Duan et al., 2015 ▶)	↑Learning and memory ↓AChE activity and quantity ↓Aβ aggregation ↑Microglia, astrocytes, neurons ↑Synaptic plasticity	ip injection (2, 20, 200 mg/kg) every day for 4 weeks	Female and male APP/PS1 mice (8-month-old, weight = 27.70 gr± 3.47 gr)	
(Lu et al., 2009b ▶)	↓Short-term memory impairment ↓Recognition memory impairment	Oral gavage (2, 20, and 200 mg/kg) 60 min before the Y-maze test and the training session of the novel object test	Male ICR mice, five weeks old	
(Bai et al., 2017 ▶)	↓Memory Impairments ↓Oxidative Stress ↓ Apoptosis ↑Synapses protection	Oral administration (100 or 200 mg/kg) once per day for eight weeks	Five months male APP/PS1 mice	
(Liu et al., 2021a ▶)	↑Dopaminergic neuronal Survival ↓Oxidative stress ↓Pro-inflammatory cytokine expression ↓Neuron death	(70, 140 and 280 mg/kg) was orally administered to mice daily for seven days	Male C57BL/6 (8 weeks old, weight 20–23 gr)	
(Lee et al., 2015a ▶)	↓Dopaminergic neuron loss ↓Glial activation in STR and SN	ip injection (1 or 10 mg/kg) for 14 days	Male C57B/6 mice (7 weeks old, weight 18–21 gr)	PD
(Lee et al., 2015b ▶)	↓Dopaminergic neuronal loss in STR and SN Not Affect Glial Activation ↓Neuronal morphological changes	ip injection of silibinin (1 and 10 mg/kg) for eight days	Male C57B/6 mice (7 weeks old, weighing 18-21 gr)	
(Liu et al., 2021b ▶)	↓Cognitive Function Deficits ↓Neuronal loss in HIP and cortex ↓Oxidative Stress	administration of intragastric (*i.g.*) 70, 140, and 280 mg/kg for 7 days	Male C57BL/6 (8 weeks old, weight 20–23 gr)	

AChE: Acetylcholinesterase; Aβ: Amyloid beta; NPCs: Neuronal precursor cells; STR: Striatum; SN: Substantia nigra; GCD: Granule cell dispersion; PFC: Prefrontal cortex; BBB: Blood Brain Barrier; PD: Parkinson’s disease

Glial cells, including astrocytes and microglia are the main mediators of neuroinflammation. Suppressing the activity of glia, especially inflammatory microglia, can play an effective role in controlling inflammation (Leonoudakis et al., 2017 ▶).Studies show that plant compounds such as resveratrol can suppress inflammation by reducing the activity of microglia (Ghazavi et al., 2020 ▶). The inflammatory response could be controlled by silibinin by regulating IL-4 (Song et al., 2016 ▶). In addition, silibinin treatment for 40 days significantly improved memory performance in the Morris water maze (MWM), Y Maze (YM), and elevated plus maze (EPM). The hippocampus receives abundant inputs from the basal forebrain cholinergic system, which plays a vital role in memory formation. Silibinin decreases acetylcholinesterase activity elevated by chronic stress and enhances acetylcholine (ACh) levels (Garikapati et al., 2018 ▶). 

**Figure 2:**
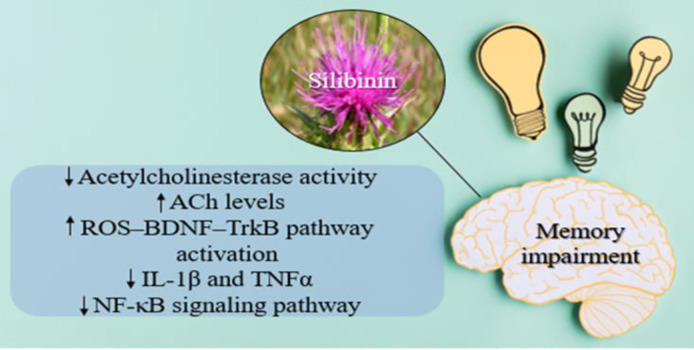
Silibinin improves memory by reducing acetylcholinesterase activity and increasing the acetylcholine amount. On the other hand, it prevents neuronal death in the hippocampus by reducing inflammation and increasing antioxidant factors. Ach: acetylcholine; ROS-BDNF-TrKB: Reactive oxygen species- Brain-derived neurotrophic factor- Tropomyosin receptor kinase B; IL-1β: Interleukin 1 beta; TNFα: Tumor necrosis factor; NF-κB: nuclear factor kappa-light-chain-enhancer of activated B cells

Brain-derived neurotrophic factor (BDNF) is a protein from the family of neurotrophins. It plays a role in important cognitive functions such as synaptic plasticity, learning, and memory in the mammalian brain (Brigadski and Leßmann, 2014 ▶; Song et al., 2016 ▶). BDNF and tropomyosin-related kinase receptor type B (TrkB) as its receptor exist in different parts of the brain (Jin, 2020 ▶). Reactive oxygen species (ROS) regulate various biological processes such as cell proliferation, differentiation, migration, and survival (Song et al., 2016 ▶). Silibinin treatment (25, 50, and 100 mg/kg) reduced inflammation. It activated the ROS–BDNF–TrkB pathway in the hippocampus. It ameliorated depressive-like behaviors by decreasing the production of IL-1β in the hippocampus and increasing IL-1β and tumor necrosis factor alpha (TNFα) in the serum of LPS-induced memory impairment rats (Song et al., 2016 ▶). Also, silibinin treatment reduced the LPS-induced loss of working memory function and anxiolytic activity; therefore, silibinin can effectively improve anxiolytic activity and cognitive deficits. A decrease in the NF-κB signaling pathway in the prefrontal cortex (PFC) by silibinin attenuates pro-inflammatory cytokines and neuroinflammation induction. Moreover, it significantly decreased the hippocampus's amyloid precursor protein (APP) expression (Joshi et al., 2014 ▶). Silibinin also improves memory impairment by regulating estrogen receptors in amyloid-beta1-42 (Aβ1-42)-injected rats (Song et al., 2016 ▶). The function of silibinin in improving memory is shown in Figure 2.

**Table 2:** Mechanisms of silibinin effect in improving cognitive disorders

**Ref.**	**Mechanisms**	**Main finding**	**Model**
(Song et al., 2016 ▶)	↓NF-κB signaling pathway ↓Expression of IL-1β and TNFα in serum ↓Production of IL-1β in HIP ↑Production IL-4 in the HIP ↓GSH-PX activity in HIP ↑ROS–BDNF–TrkB pathway in HIP	↓Neuroinflammation ↓Neuronal Loss	Stress and Memory Impairment
(Joshi et al., 2014 ▶)	↑ACh level in HIP and PFC ↓AChE activity in HIP and PFC ↑Mitochondrial function and integrity ↑The activity of mitochondrial complex enzymes ↓Expression of Aβ in HIP ↓Expression of NF-κB in PFC ↓Expression of APP in HIP	↓Stress oxidative in the hippocampus and PFC	
(Tota et al., 2011 ▶)	↓ AChE activity ↓AChE mRNA expression	↓Memory impairment	
(Lee et al., 2020 ▶)	↑5-HT level in the HIP ↑5-HT in the Amg ↓5-HIAA levels in HIP ↑TPH-1 expression in HIP ↓ plasma COR levels	↓depression ↓anxiety	PTSD
(Garikapati et al., 2018 ▶)	↑ protein content ↑ SOD activity ↑GSH activity ↑AchE activity ↓TNF α	↑Memory function	chronic stress
(Lu et al., 2010 ▶)	↑Dopamine in PFC ↑Serotonin in HIP	↓learning and memory impairment	Addiction
(Wei et al. 2022 ▶)	↑expression of HO-1 protein ↓apoptosis ↓ROS ↑expression of ALDH2, GSH, ADH3 ↓ expression of phosphorylated GSK3β (Y216) ↓expression of tau protein hyperphosphorylation of p-SER-396 and p-SER-404 in HIP	↑Spontaneous alternation behavior ↑Spatial memory	cognitive impairment
(Gu et al., 2018 ▶)	No effect on plasma lipid levels ↑CBF ↑Number of Open Microvessels in HIP ↓rough inner surface of microvessels ↓hippocampal neuronal damages ↑claudin-5, occluding, ZO-1 expression	↑Learning and memory ↑BBB integrity	Dementia induced-familial hypercholesterolemia
(Wang et al., 2012 ▶)	↑Expression of pAkt, pmTOR, HIF-1˛ Bcl-2 ↓Expression of Bax, NF-κB	↓Brain edema ↓Infarct volume	Ischemic stroke
(Xie et al., 2014 ▶)	↓ROS production ↑Mitochondrial membrane potential (MMP) ↑LKB1–AMPK–ACC signaling in neuronal cells	↓Neuronal cell death ↓Neuronal cells apoptosis	
(Fernandes et al., 2018 ▶)	↓GFAP expression ↑GSH levels ↓MDA formation ↑SOD activity ↑catalase activity	↓Astrocytes activation ↓Oxidative stress ↓Nitrosative stress	Oxidative-nitrosative damage and astrocyte activation
(Kim et al., 2017 ▶)	↓mTORC1 activation ↓c-caspase-3, c-PARP-1 ↓LC3B, LC3-II expression ↓TNF-α, and IL-1β levels	↑Memory function ↓Granule cell dispersion (GCD) ↓Neuronal cell loss ↓Apoptosis ↓Autophagy ↓Neuroinflammation	Epileptic seizures
(Jin et al., 2016 ▶)	↓Levels of Iba1 in the cerebral cortex ↓IL-6 in the cerebral cortex ↓IL-4 in the cerebral cortex ↓iNOS and COX-2 in HIP ↓Phosphorylation of JNK and p38 ↓Phosphorylation of ERK1/2	↓Microglial activation ↓Neuroinflammation ↓Neuronal death	Alzheimer’s disease (AD)
(Song et al. 2017 ▶)	↑Expression of IL-4 ↓Expression of IL-1β macrophage lineage and Th2 cells Inhibition of NF-κB pathway Suppression of COX-2 and iNOS ↑GSH levels ↓MDA levels Downregulation of p53	↓Neuroinflammation ↓Stress oxidative ↑Autophagy	
(Shen et al., 2019 ▶)	↓Diversity and abundances of gut bacterial species associated with AD ↓D -alanine metabolism in the brain	↓Amyloid plaque in the hippocampus	
(Duan et al., 2015 ▶)	↑Iba1, GFAP, NeuN, DCX ↑Number and density of dendritic spines in HIP	↓AChE activity and quantity ↓Aβ aggregation ↑BDNF ↑Microglia, astrocytes, neurons, NPCs ↑Synaptic plasticity	
(Lu et al., 2009b ▶)	↓MDA levels ↑GSH levels	↓Oxidative events	
(Bai et al., 2017 ▶)	↓MDA expression ↑SOD and GSH expression ↓ Caspase-3 activity ↑Synaptophysin, PSD95 expression	↓Oxidative Stress ↓ Apoptosis ↑Synapses protection	
(Liu et al., 2021a ▶)	↑Tyrosine hydroxylase in dopaminergic neurons ↓MDA and ↑GSH-PX activity ↓Levels of NLRP3, caspase-1, and IL-1β in STR ↓Expression of α -syn in the striatum ↑Mitophagy (↑expression of Parkin, PINK1 - ↑The ratio of LC3 II/LC3 I)	↑Dopaminergic neuronal Survival ↓Stress oxidative in the striatum ↓Pro-inflammatory cytokine expression ↓Neuron death	PD
(Lee et al., 2015a ▶)	↑TH protein expression in STR and SN ↓GFAP expression ↓ERK and JNK signaling	↓Dopaminergic neuron loss ↓Glial activation in STR and SN	
(Lee et al., 2015b ▶)	↑Tyrosine hydroxylase ↓Loss of F-actin Prevention of MMPs disruption	↓Dopaminergic neuronal loss in STR and SN ↓Neuronal morphological changes ↑Mitochondrial Stabilization	
(Liu et al., 2021b ▶)	↑Bcl-2 ↓Bax, cleaved-caspase-3, cleaved-caspase-8, cleaved caspase-9 ↓αSynuclein aggregation in DG, CA1, CA3 ↓MDA level	↓Neuronal loss in the hippocampus and cortex ↓Apoptosis ↓Oxidative Stress ↓Mitochondrial dynamic disorder	

5-HIAA: 5-hydroxyindoleacetic acid; TPH-1: tryptophan hydroxylase-1; IL-4: Interleukin-4; IL-6:  Interleukin-6; IL-1β: Interleukin 1 beta; Th2 cells: T helper cells; NF-κB: Nuclear factor kappa-light-chain-enhancer of activated B cells; iNOS: Inducible nitric oxide synthase; GSH: Glutathione; GSH-PX: Glutathione peroxidase; MDA: Malondialdehyde; Iba1: Ionized calcium binding adaptor molecule 1; JNK: Jun N-terminal kinase; ERK1/2: Extracellular signalregulated protein kinase; GFAP: Glial fibrillary acidic protein; DCX: Doublecortin; BDNF: Brain-derived neurotrophic factor; Bcl-2: B-cell lymphoma 2; BAX: Bcl-2-associated X protein; NLRP3: NLR family pyrin domain containing 3; PINK1: PTEN-induced kinase1; MMP: Matrix metalloproteinase; HIF-1: Hypoxia-inducible factor-1; ROS: Reactive oxygen species; AMPK: AMP-Activated protein kinase; LKB1: Liver kinase B1; ACC: Acetyl-CoA Carboxylase; PD: Parkinson’s disease; TNF-α: Tumor necrosis factor alpha; HIP: Hippocampus; ROS–BDNF–TrkB: Reactive oxygen species- Brain-derived neurotrophic factor- Tropomyosin receptor kinase B; Ach: Acetylcholine; AchE: Acetylcholine Acetylcholinesterase; COR: cortisol; COX-2: Cyclooxygenase-2; AD: alzheimer disease; 5-HT: 5-hydroxytryptamine; STR:striatum; SN: substantia nigra; DG: dentate gyrus; ERK: extracellular signal-regulated kinase; LC3 II/LC3 I: light chain3 II/ light chain3 I; α -syn: α-synuclein; PSD-95: Postsynaptic density protein 95; SOD: Superoxide dismutase; PARP1: poly (ADP-ribose) polymerase-1; CBF: Cerebral blood flow; GSK3β: Glycogen synthase kinase-3β; PFC: prefrontal cortex; ALDH2: aldehyde dehydrogenase-2; ADH3: formaldehyde dehydrogenase; HO-1: Heme oxygenase

**Effects of silibinin on cognitive dysfunction, inflammatory responses, oxidative stress, and autophagy in the non-demented diseases**

In everyday life, every person may temporarily suffer from various cognitive disorders. However, it becomes a clinical concern when this disorder becomes severe, persistent, or progressive due to factors such as stroke, drug poisoning, and alcoholism (Koliatsos, 2016 ▶).

**Figure 3:**
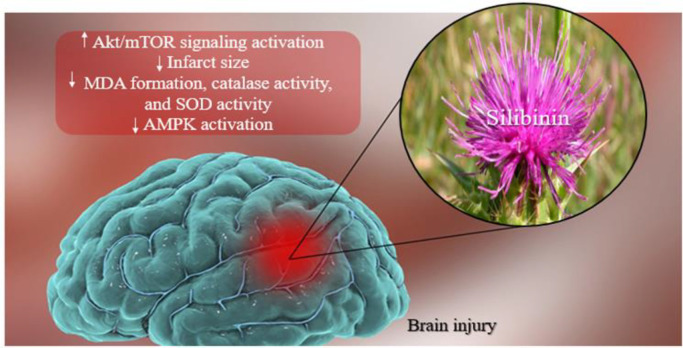
Silibinin prevents the actions of oxidative stress, apoptosis, and autophagy pathways in brain damage. AKt/mTOR: protein kinase B/ mammalian target of rapamycin; MDA: Malondialdehyde; SOD: Superoxide dismutase; AMPK: AMP-Activated protein kinase

Cognitive impairment is common after a stroke. Almost one-third of patients experience various degrees of cognitive impairment, including problems of attention, memory, language, and orientation, within one year after the onset of stroke (Al-Qazzaz et al., 2014 ▶). One of the reasons for the occurrence of cognitive impairment after a stroke is the increase in neurotoxicity which spreads throughout the brain and affects other parts (Vafaee et al., 2014 ▶; Ghazavi et al., 2020 ▶). Following neurotoxicity, pro-inflammatory responses, apoptosis, oxidative stress, and autophagy occur (Shivani et al., 2017 ▶). mTOR is a serine/threonine protein kinase that contributes to cytoskeleton growth and formation. It is also a vital regulator of the early stage of autophagy. During ischemic stroke, mTOR is involved in regulating autophagy with several signaling pathway components, such as protein kinase B/ mammalian target of rapamycin (Akt/mTOR) (Qin et al., 2022 ▶). Activation of the Akt/mTOR pathway helps promote neovascularization which can lead to a reduction in infarct size after stroke. (Hwang and Kim, 2011 ▶). As shown in Figure 3, some studies have shown the regulatory effect of silibinin after an ischemic neuronal injury that may be associated with the activation of Akt/mTOR signaling. Silibinin also reduced brain edema and infarct size 24 and 72 hr after ischemia, which led to post-stroke cognitive dysfunction amelioration (Wang et al., 2012 ▶).

In many diseases, such as stroke, epilepsy, and stress, ROS levels increase to toxic levels and cause cellular damage through lipid peroxidation. Neurons are vulnerable due to their high oxidative metabolism and fewer antioxidant enzymes (Olmez and Ozyurt, 2012 ▶). Silibinin treatment (12.5, 25, and 50 μM) significantly reduced nitrite release, intracellular ROS generation, MDA formation. But SOD and catalase activity increased by silibinin. It also ameliorated the reduced glutathione (GSH) level indicating a reduction in intracellular oxidative stress revealing a critical antioxidant role in the maintenance of intracellular oxidative stress and subsequent neuroprotection (Fernandes et al., 2018 ▶). Silibinin can inhibit the development of inflammatory reactions in the human mast cells-1 (HMC-1) by blocking the NF-κB signaling pathway (Kim et al., 2013 ▶). In addition, *in vitro* studies have shown that silibinin activates vitagenes, responsible for synthesizing protective molecules, such as heat shock proteins (HSPs), thioredoxin, and sirtuins, and may also protect against oxidative stress (Surai, 2015 ▶; Kadoglou et al., 2022 ▶). In another study, the administration of silibinin (20 mg/kg/day, i.p. for four weeks) reduced oxidative stress and DNA damage in the brain of db/db mice, and the involved pathway in the neuroprotective effect was heme oxygenase-1 (Marrazzo et al., 2011 ▶; Oh, 2016 ▶). In another animal study, the silibinin-mediated neuroprotective effect significantly inhibited oxygen and glucose deprivation (OGD)/re-oxygenation-induced ROS production and AMP-Activated protein kinase (AMPK) activation (Xie et al., 2014 ▶). 

Epilepsy is one of the neurological diseases that cause impairment in cognitive function and hurts lives (Stafstrom and Carmant, 2015 ▶; Anaeigoudari et al., 2016 ▶). Antiepileptic drugs are used to treat and control epileptic seizures. However, these drugs have side effects in some patients that can hinder the treatment. Therefore, there is a need to improve the treatment of epilepsy. For a long time, herbal medicines have been considered for treating epilepsy (Aghdash, 2020 ▶). In an animal model, pretreatment of silibinin (50, 100, and 200 mg/kg) in kainic acid (KA) - induced status epilepticus (SE) in mice showed a significant neuroprotective effect that might be due to reducing the expression of inflammatory cytokines IL-1β and TNF-α. Its molecular mechanism may be associated with the cleaved caspase-3 protein, poly (ADP-ribose) polymerase-1 (PARP1) cleavage, and inhibition of anti-apoptotic effects such as light chain 3B (LC3B) expression and light chain 3-II (LC3-II) levels, inhibition of Granule cell dispersion (GCD) via suppression of mTORC1 activation (Kim et al., 2017 ▶).

**Effects of silibinin on cognitive dysfunction, inflammation, and oxidative stress in the demented diseases**

With the improvement of global health, people reach old age, increasing the incidence of neurodegenerative diseases. Alzheimer's disease (AD) is one of the most common cognitive diseases of the elderly worldwide. It is a progressive and inflammatory neurodegenerative disease that causes cognition disorders. Its pathological features are marked by the accumulation of amyloid-β (Aβ) plaques, hyperphosphorylation of tau proteins, activation of microglia, and the death of neurons (Cai et al., 2014 ▶; Bolós et al., 2017 ▶; Shirzad et al., 2020 ▶).

Vital elements in the emergence of AD include Aβ production and neuroinflammation. There is a vicious cycle of inflammation among Aβ accumulation, activation of microglia and astrocytes (AST), and inflammation mediators (Cai et al., 2014 ▶; Domingues et al., 2017 ▶). 

A study reported that silibinin significantly protected Aβ25-35-induced memory deficits in the Morris water maze, memory flexibility tests, and novel object recognition. In addition, this study reported that silibinin exerted a protective effect by increasing autophagy levels and anti-inflammatory action (Song et al., 2017 ▶). p53 has an important role in neurodegenerative diseases and was elevated in the brain of sporadic AD (Ohyagi et al., 2005 ▶). Silibinin reduces p53 levels, suppressing inflammatory response (Song et al., 2017 ▶). In the senescence-accelerated mouse (SAMP8)-induced AD male mice, silibinin (100 or 200 mg/kg) treatment effectively attenuated and reduced microglial activation and prevented the progression of the neuroinflammatory reaction in SAMP8 mice (Jin et al., 2016 ▶).

With the increase in age, the decrease in estrogen levels, and the occurrence of many changes in the expression and signaling of estrogen receptors (ERs), cognitive disorders and the percentage of cerebral dementia increase in women (Wang et al., 2016 ▶). Silibinin acts as an agonist for ERβ, and its injection into the hippocampal CA1 region (25, 50, and 100 mg/kg) causes a neuroprotective effect via increasing cognitive function and ERs expression levels in the hippocampus of Aβ1−42-injected rats (Song et al., 2018 ▶). Cholinergic neurotransmission plays a role in cognitive dysfunction in AD, so acetylcholinesterase (AChE) inhibitors are used in many treatments (Pérez-Gómez Moreta et al., 2021 ▶). A recent study showed that treatment with silibinin significantly reduced Aβ aggregation and quantity of AChE (Shen et al., 2019 ▶). 

Moreover, increase in neurogenesis, synaptic protection, gliogenesis, and a significant increase in dendritic spines in the brain (Duan et al., 2015 ▶). Silibinin has anti-inflammatory effects and might also lead to the up-regulation of IL-4 production in the hippocampus of Aβ25-35-injected rats (Reale et al., 2006 ▶; Song et al., 2017 ▶). In Aβ25–35 protein-induced AD mice, silibinin (2, 20, and 200 mg/kg) prevented the accumulation of markers of lipid peroxidation, such as MDA. In addition, increased cellular glutathione (GSH) prevented oxidative damage in the hippocampus (Lu et al., 2009b ▶). Several studies showed that the GSH system might be activated as a response to oxidative stress in AD (Aksenov and Markesbery, 2001 ▶). Based on the data presented in previous studies, silibinin was able to increase the level of GSH and decrease the level of MDA in mice injected with Aβ25-35 (Bai et al., 2017 ▶; Song et al., 2017 ▶).

Silibinin in the streptozotocin (STZ) rat AD model significantly protected learning and memory via inhibition of the hyperphosphorylation of tau protein. It could increase the expression of insulin receptor (IR) and insulin-like growth factor receptor 1(IGF-1R) and reverse impaired insulin signaling pathway (Liu et al., 2020 ▶). The administration of STZ twice in mice caused a persistent memory deficit, and silibinin treatment improved STZ-induced memory deficits (Tota et al., 2011 ▶). Silibinin ameliorates cognitive dysfunction caused by inhibiting the expression of phosphorylated glycogen synthase kinase-3β (GSK-3β), promoting the nuclear transfer of NRF2 (Wei et al., 2022 ▶). The other study included old people with minor "cognitive impairment" (MCI). After sixteen weeks of treatment with silibinin, overall cognitive function, mainly memory function and verbal learning were significantly improved compared to the placebo group (Hussain et al., 2022 ▶). Silibinin ameliorated memory impairment induced by Aβ25-35; maybe due to the blocking inflammatory responses and oxidative stress in the hippocampus and amygdala (Lu et al., 2009a ▶).

Parkinson’s disease (PD) is associated with chronic inflammation, dopaminergic neuronal loss, oxidative stress, aging, mitochondrial dysfunction, and clinical symptoms that include tremors, rigidity, and bradykinesia (Wang and Michaelis, 2010 ▶). About 500 distinct DNA variants in five disease genes related to Parkinson's disease have been identified. The most important of which are α-synuclein (SNCA), parkin (PARK2), PTEN-induced putative kinase 1 (PINK1), DJ-1 (PARK7), and Leucine-rich repeat kinase 2 (LRRK2) (Nuytemans et al., 2010 ▶). These genes are also involved in oxidative stress response, the ubiquitin protein degradation pathway, and mitochondrial function. So, one of the proposed treatments for Parkinson's disease is a natural antioxidant like silibinin (Chang and Chen, 2020 ▶). Silibinin's efficacy in treating PD increases mitophagy mediators, including PINK1 (PTEN-induced kinase 1) and parkin, which promote mitophagy to remove injured mitochondria. In addition an increase in the ratio of LC3 II/I (microtubule-associated protein light chain 3) that indicates augmented mitophagy. Thus silibinin attenuates α-synuclein toxicity and oxidative stress by promoting mitophagy (Liu et al., 2021a ▶). A study reported that silibinin intraperitoneal (ip) injection (1 or 10 mg/kg) in acute 1-Methyl-4-phenyl-1,2,3,6-tetrahydropyridine (MPTP)-induced PD model decreased glial activations and dopaminergic neuronal loss in the substantia nigra. In addition, a 2-week silibinin pretreatment (1 or 10 mg/kg per day) effectively inhibited MPTP-induced neuroinflammation via attenuating glial fibrillary acidic protein (GFAP) activation in astrocytes (Lee et al., 2015a ▶). Extracellular signal-regulated kinase (ERK) and c-Jun N-terminal kinase (JNK) are known as mitogen-activated protein kinases (MAPKs). They are activated by many stimuli, such as mitogenic signals, cellular stress, cytokines, and antigen receptor ligation. The ERK and JNK pathways are known to play significant regulatory roles in cellular processes associated with PD (Sakata et al., 1999 ▶; Anderson et al., 2007 ▶; Peterson and Flood, 2012 ▶). Previous studies showed that the expression levels of phosphorylated JNK were reduced by silibinin (Bai et al., 2017 ▶; Jin et al., 2016 ▶). 

In an MPTP-induced PD model, ip injection of silibinin treatment (1 and 10 mg/kg) effectively attenuated motor deficits and dopaminergic neuronal loss. Low concentrations of silibinin (<10 µM) failed to attenuate membrane palmitoylated protein 1 (MPP1)- induced ROS production in primary cultured neurons, but these doses effectively protected neurons against MPP1-induced toxicity. It protected neurons by reducing ROS generation levels at a high concentration (50 µM) (Lee et al., 2015b ▶). 

**Figure 4:**
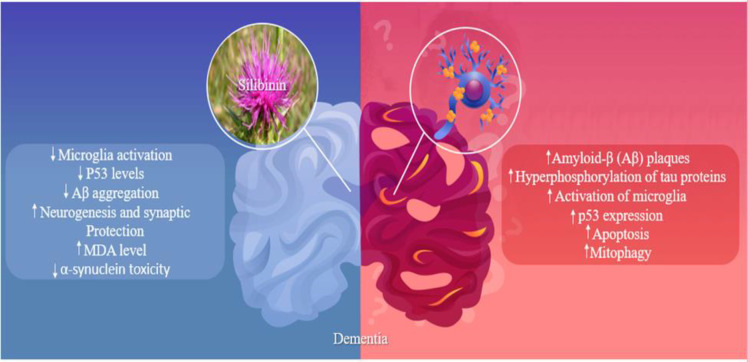
In cerebral dementia, increased inflammation due to overexpression of glia and p53 causes apoptosis and cell death. Silibinin has been able to reduce these factors significantly. MDA: Malondialdehyde

In an *in vitro* assay, MPTP injection caused damage to hippocampal cells by inducing α-synuclein aggregation, elevating oxidative stress, and disturbing mitochondrial dynamic equilibrium. Silibinin treatment (280 mg/kg) significantly reduced the activity of SOD and MDA level, suggesting that silibinin reduces oxidative stress in PD mice. In addition, administration of silibinin (70, 140, and 280 mg/kg) in MPTP-injected experimental model of PD significantly rescuedapoptosis via decreasing the protein levels of Bax, cleaved-caspase-3, cleaved-caspase-8, and cleaved-caspase-9 and increasing B-cell lymphoma 2 (Bcl-2). Furthermore, increased mitochondrial membrane potential (MMP) indicated that silibinin had protective effects on mitochondrial disorders. Furthermore, it might explain how silibinin improved cognitive dysfunction (Liu et al., 2021b ▶). Figure 4 briefly shows the pathways affected by silibinin in cerebral dementia. 

**Effects of silibinin on cognitive impairments in addiction and neurotoxicity**

Addictive drugs affect some cognitive functions by affecting brain regions such as the prefrontal cortex, hippocampus, amygdala, and striatum. These drugs affect dopamine and serotonin pathways in the brain reward system and cause cognitive disorders (Adinoff, 2004 ▶; Arias-Carrián et al., 2010 ▶; Gould, 2010 ▶).

Some drugs, including morphine, heroin, methamphetamine, and cocaine, cause hippocampus-dependent memory impairments (Goodman and Packard, 2016 ▶). Methamphetamine decreases dopamine in the prefrontal cortex and serotonin in the hippocampus and affects dopaminergic and serotonergic terminals in the limbic system (Lu et al., 2010 ▶). Some studies have reported that both pathways play a role in the recognition memory impairment (Sehatpour et al., 2008 ▶). Although silibinin does not affect recognition memory in normal conditions, it reduced the recognition memory impairment in methamphetamine (METH)-injected mice by regulating the dopamine and serotonin content of the prefrontal cortex and hippocampus. These effects of silibinin were exerted through a decrease in homovanillic acid/dopamine (HVA/DA) ratio and Dihydroxyphenylacetic acid + Homovanillic acid/Dopamine (DOPAC + HVA/DA) ratio in the prefrontal cortex and 5-hydroxyindoleacetic acid/5-hydroxytryptamine (5-HIAA/5-HT) ratio in the hippocampus (Lu et al., 2010 ▶).

The blood-brain barrier (BBB) has tight junctions between endothelial cells of the brain capillary and prevents various compounds' permeation to the brain. However, some compounds can permeate the BBB and cause brain dysfunctions such as cognitive disorders through the induction of neurotoxicity (Pardridge, 2012 ▶). Silibinin reduced formaldehyde-induced neurotoxicity via activation of the NRF2 pathway as an essential pathway of oxidative stress reduction without affecting NRF2 protein expression in the hippocampus. It caused improvement in working memory impairment and spatial learning and memory impairment. Also, the administration of silibinin increased formaldehyde-degrading enzymes, GSH, and aldehyde dehydrogenase 2 (ALDH2) in formaldehyde-induced rats (Wei et al., 2022 ▶).

**Effects of silibinin on anxiety and depression**

The laboratory models showed increased anxiety and depression behaviors due to decreasing serotonin in the hippocampus and amygdala. Tryptophan hydroxylase-1 (TPH-1) is a rate-limiting reaction in serotonin biosynthesis. Some studies showed the possible role of the decline of TPH-1 in psychiatric conditions. TPH-1 expression decline causes anxiety and depression-like behaviors through serotonin production dysfunction in the hippocampus and amygdala (Nakamura et al., 2006 ▶; Lee et al., 2020 ▶). Silibinin reduced 5-hydroxyindoleacetic acid (5-HIAA), the primary metabolite of serotonin, when serotonin is broken down in the liver (Gedde-Dahl et al., 2013 ▶).

## Discussion

Based on the results of the present project, silibinin has ameliorative effects on cognitive functions by various mechanisms. Inflammation is an important and common occurrence of many neurological disorders, and NF-κB is one of its signaling pathways. NF-κB includes a family of transcription factors that cause gene expression related to immune and inflammation responses (Liu et al., 2017 ▶). Silibinin leads to decreased inflammation arising from dementia and non-dementia diseases, stress, memory impairments, and oxidative damage via inhibition of NF-κB signaling pathway and affecting Akt/mTOR signaling pathway and improves cognition functions (Hwang and Kim, 2011 ▶).

Although apoptosis occurs in cell physiological conditions and is crucial for normal cell turnover, improper apoptosis occurs in many neurological disorders such as PD, AD, and ischemic stroke, causing the loss of neurons, and accelerating cognitive disorders (Elmore, 2007 ▶; Liu et al., 2020 ▶). The effect of silibinin on pathological apoptosis is an increase in Bcl2 expression and a decrease in Bax expression and caspase activation of the apoptosis pathway (Xie et al., 2014 ▶; Bai et al., 2017 ▶; Liu et al., 2020 ▶).

Silibinin modulates serotonin synthesis through its effect on TPH-1 function; therefore, the amount of serotonin's primary metabolite, 5-HIAA, decreases in silibinin treatment. In this way, it causes the improvement of cognitive disorders caused by serotonin deficiency, such as anxiety (Gedde-Dahl et al., 2013 ▶). As an agonist of estrogen receptors in CA1 of the hippocampus, silibinin plays a role in cognitive function improvement (Wang et al., 2016 ▶). It seems that the effect of silibinin on learning and memory in different neurological disorders is through the activation of ROS–BDNF–TrkB pathway in the hippocampus, an increase of dendritic spines in the brain, inhibition of hyperphosphorylation of tau protein, and an increase in the expression of IR and IGF-1R, blocking of inflammatory responses and oxidative stress in the hippocampus and amygdala, and a decrease in HVA/DA ratio and DOPAC + HVA/DA ratio in the prefrontal cortex and 5-HIAA/5-HT ratio in the hippocampus (Lu et al., 2009a ▶, 2010; Duan et al., 2015 ▶; Song et al., 2016 ▶; Liu et al., 2020 ▶).

Oxidative stress occurs in many neurological diseases, damages the cells of the nervous system, and causes cognitive disorders. By excessive production of ROS in cells, and given that cells cannot remove excessive ROS, it accumulates in the cell (Uttara et al., 2009 ▶; Patel, 2016 ▶; Pizzino et al., 2017 ▶). ROS level is low in cells under physiological conditions and causes normal activation of many processes such as differentiation, apoptosis, immunity, and transcription factors activation (Rajendran et al., 2014 ▶). However, ROS levels increase in pathological conditions and damage essential cell structures such as lipids, proteins, and nucleic acids (Wu et al., 2013 ▶). Silibinin causes oxidative stress reduction by decreasing MDA expression and increasing GSH and SOD expression (Tota et al., 2011 ▶; Liu et al., 2021b ▶).

The results of various studies show that silibinin is an effective agent that has preventive and therapeutic effects on cognitive disorders. Silibinin improves learning and memory disorders and can reduce depression, anxiety, and other cognitive disorders caused by addiction, neurotoxicity, dementia, and non-dementia diseases through effects on various mechanisms, including inflammatory responses, programmed cell death, oxidative stress, and modulation of serotonin synthesis. However, given that many activated pathways in various cognitive disorders are common, further studies are needed to understand better common mechanisms by which silibinin affects cognitive functions.

## Conflicts of interest

The authors have declared that there is no conflict of interest.
